# Modelling Lassa virus dynamics in West African *Mastomys natalensis* and the impact of human activities

**DOI:** 10.1098/rsif.2024.0106

**Published:** 2024-07-24

**Authors:** Reju Sam John, Hammed Olawale Fatoyinbo, David T. S. Hayman

**Affiliations:** ^1^ Massey University, Private Bag, 11 222, Palmerston North 4442, New Zealand

**Keywords:** Lassa fever, time-average basic reproductive number, ${\tilde{R}}_{p}$, climate change, land use change

## Abstract

Lassa fever is a West African rodent-borne viral haemorrhagic fever that kills thousands of people a year, with 100 000 to 300 000 people a year probably infected by Lassa virus (LASV). The main reservoir of LASV is the Natal multimammate mouse, *Mastomys natalensis*. There is reported asynchrony between peak infection in the rodent population and peak Lassa fever risk among people, probably owing to differing seasonal contact rates. Here, we developed a susceptible-infected-recovered ( SIR )-based model of LASV dynamics in its rodent host, *M. natalensis*, with a persistently infected class and seasonal birthing to test the impact of changes to seasonal birthing in the future owing to climate and land use change. Our simulations suggest shifting rodent birthing timing and synchrony will alter the peak of viral prevalence, changing risk to people, with viral dynamics mainly stable in adults and varying in the young, but with more infected individuals. We calculate the time-average basic reproductive number, ${\tilde{R}}_{p}$ , for this infectious disease system with periodic changes to population sizes owing to birthing using a time-average method and with a sensitivity analysis show four key parameters: carrying capacity, adult mortality, the transmission parameter among adults and additional disease-induced mortality impact the maintenance of LASV in *M. natalensis* most, with carrying capacity and adult mortality potentially changeable owing to human activities and interventions.

## 1. Introduction

Lassa fever is a viral haemorrhagic fever (VHF) caused by Lassa virus (LASV, species *Mammarenavirus lassaense*). LASV is endemic in West Africa. Approximately 100 000 to 300 000 people may be infected annually, with a fatality rate of 1−2% of cases, though reported case fatality rates can be much higher [1,2]. The main reservoir of LASV, the species in which the virus persists at the population level, is the Natal multimammate mouse, *Mastomys natalensis*, though numerous species have contact with LASV and may become infected [1,3]. *Mastomys natalensis* is a rodent species that is not only infected with LASV, but lives in villages and agricultural habitats throughout West Africa [4,5]. Humans probably become infected through the ingestion of food or water contaminated with LASV-positive droppings and urine, the inhalation of aerosolized virus particles or sometimes the direct consumption of LASV infected mice [4,6]. Human-to-human transmission is rare, though reported from households and hospitals, so the main source of infection in people is zoonotic (animal to human) [7–9]. The first-in-human phase 1 vaccine trial results have been recently published and are promising [10], but there is currently no licensed vaccine or effective treatment, and, given the high case fatality and evidence of human-to-human transmission, the World Health Organization includes LASV as a priority pathogen of epidemic potential.

The natural LASV host, *M. natalensis* is distributed all over sub-Saharan Africa, where it also hosts other arenaviruses [11], yet while suitable habitat exists that might allow the ecological opportunity for viral spread among regions, different sub-taxa among *M. natalensis* have different ranges, and these support intrinsic barriers among hosts, preventing infection with different viruses, thus explaining why human cases of LASV are limited to West Africa [12].

The risk of LASV infection is seasonal [4,13]. However, there has been a reported disconnect (asynchrony) between the dynamics of hosts [14] and infection in hosts [4] and human risk (though possible exceptions exist [15], supported by some recent modelling work [16]). Most human LASV infections occur in the dry season (probably owing to contact), yet viral prevalence and rodent breeding are higher in the rainy season [4]. Prevention of LASV infection is currently effectively limited to hygiene and rodent control. However, recent studies have shown some mouse control approaches might alter the dynamics and even lead to increases in host infection, and therefore human risk, following population recovery [17], a phenomenon also possibly observed after efforts to cull bats infected with VHF-causing marburgviruses [18]. Together, these findings suggest rodent behaviours and their responses to the environment may play a key role in determining human infection and disease risk [4,14,16]. This risk might change owing to land use and climate change, along with human population growth in West Africa [19,20].

Few mathematical models have examined the seasonal dynamics of LASV and incorporated rodent dynamics, with recent models aiming to fit spillover models to data and understand changes in risk, including with seasonal variation and how rodent control might impact the risk of LASV infection among people [13,16,21–23]. The historic gap is partly probably owing to the gaps in knowledge and data available on *M. natalensis* populations and LASV in West Africa, despite the obvious public health impacts [1] and the species importance as a crop pest [24–27]. However, this gap needs filling. *Mastomys natalensis* is generalist species, feeding on various available resources depending on the season and the habitat [25,28], including crops such as maize [29], causing population densities to coincide with resource availability [27]. West Africa and Africa in general are going through substantial land use change, potentially changing habitats and disease risk [30] and will be impacted by climate change [31], all impacting resource availability for *M. natalensis* and contact rates between them and people [19].

Here, we investigate the seasonal dynamics of LASV in a model *M. natalensis* population to further help understand how the host population dynamics might affect viral persistence in the population, its dynamics, and how changing host dynamics, such as in response to land use or climate change, might impact infection dynamics.

## 2. Methods

**Figure 1.**
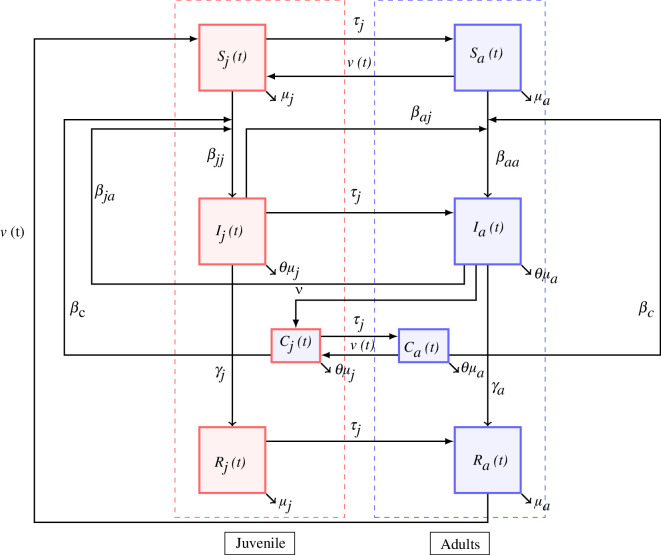
Model structure.

We follow recent arenavirus susceptible–infected–recovered ( SIR ) models in *M. natalensis* [23,32] based on field observations and experimental infection data, occasionally using other rodent-mammarenavirus data if *M. natalensis*-LASV data are not available (table 1). The SIR model structure incorporates demography (births; ν and deaths; μ ). Field and experimental data suggest some individuals can be chronically (persistently) infected and infectious, so we include a chronic class (*C*) [23,37,38], and that while most infection is horizontal [39], there is evidence of vertical transmission (or pseudo-vertical, to suckling young) in approximately 10% of young (the viral prevalence in pregnant females [14]), with neonatally infected animals acquiring chronic infection [14,40,41]. Therefore, we include age structure with adult ( ${,}_{a}$ ) and juvenile classes ( ${,}_{j}$ ). As all neonatal *M. natalensis* infected with LASV in an experimental study became chronically infected with simultaneous development of antibodies [40], and the evidence of vertical transmission in approximately 10% of young matches the viral prevalence in pregnant females [14], we assume all young born to infected mothers become chronically infected, leading to a different model structure to [23]. While the impact of infection on *M. natalensis* is not obvious [37], long-term studies suggest there may be an additional disease-induced mortality (approximately 5%) [34], which we include as θ , i.e. θ×μX=1.05×μX . Further, following [42] whose work supports LASV transmission being density-dependent transmission, we model transmission as density- and not frequency-dependent transmission, or a mixture of the two [43]. Unlike [23] we do not include maternally derived antibodies or an exposed class and discuss these in the §4. The full SICR model is shown in figure 1 and equation (2.1).

**Table 1.** Parameters and variables.

term	description	values	units	source/comments
ν(t)	birth rate	$\alpha_{0}+ke^{\left(-s\;cos^{2}(\pi t-\varphi )\right)}$	per female day ${,}^{-1}$	our calculation
t	time	1 to 365	day	NA
$\alpha_{0}$	baseline birth rate	2.77/30	per female day ${,}^{-1}$	our calculation
k	scaling factor	4.51/30	day ${,}^{-1}$	our calculation
s	bandwidth (time span of the birth pulse)	31.021	days	our calculation
φ	phase shift	0.58	radians	our calculation
ρ	proportion of young born infected	0.1		[14]
$\mu_{j}$	death rate of juvenile	0.013	per capita day ${,}^{-1}$	[32]
$\mu_{a}$	death rate of adult	0.02	per capita day ${,}^{-1}$	[33]
θ	additional disease-induced mortality	1.05		[34]
$\beta_{jj}$	juvenile-to-juvenile transmission rate	1.45e−3	per capita day ${,}^{-1}$	[35]
$\beta_{ja}$	juvenile-to-adult transmission rate	1.45e−3	per capita day ${,}^{-1}$	[35]
$\beta_{aa}$	adult-to-adult transmission rate	1.45e−3	per capita day ${,}^{-1}$	[35]
$\beta_{c}$	transmission rate from chronic class	1.45e−3/2	per capita day ${,}^{-1}$	our assumption; $\beta_{xx}/2$
$\gamma_{j}$	recovery rate of juvenile rodents	0.0457	day ${,}^{-1}$	[35]
$\gamma_{a}$	recovery rate of adult rodents	0.0457	day ${,}^{-1}$	[35]
$\tau_{j}$	ageing rate of juvenile to adult rodents	0.0166	day ${,}^{-1}$	[36]
K	carrying capacity	1000		


(2.1) $$\begin{array}{rl} \frac{dS_{j}}{dt} & =\nu (t)(S_{a}+R_{a}+(1-\rho )(I_{a}+C_{a}))-(\beta_{ja}I_{a}+\beta_{jj}I_{j}+\beta_{c}(C_{j}+C_{a}))S_{j}-\tau_{j}S_{j}-\mu_{j}S_{j}\left(\frac{N}{K}\right)\\ \frac{dI_{j}}{dt} & =(\beta_{ja}I_{a}+\beta_{jj}I_{j}+\beta_{c}(C_{j}+C_{a}))S_{j}-\tau_{j}I_{j}-\gamma_{j}I_{j}-\theta \mu_{j}I_{j}\left(\frac{N}{K}\right)\\ \frac{dC_{j}}{dt} & =\nu (t)(\rho (I_{a}+C_{a}))-\tau_{j}C_{j}-\theta \mu_{j}C_{j}\left(\frac{N}{K}\right)\\ \frac{dR_{j}}{dt} & =\gamma_{j}I_{j}-\tau_{j}R_{j}-\mu_{j}R_{j}\left(\frac{N}{K}\right)\\ \frac{dS_{a}}{dt} & =\tau_{j}S_{j}-\left(\beta_{aa}I_{a}+\beta_{ja}I_{j}+\beta_{c}(C_{a}+C_{j})\right)S_{a}-\mu_{a}S_{a}\left(\frac{N}{K}\right)\\ \frac{dI_{a}}{dt} & =\tau_{j}I_{j}+\left(\beta_{aa}I_{a}+\beta_{ja}I_{j}+\beta_{c}(C_{a}+C_{j})\right)S_{a}-\gamma_{a}I_{a}-\theta \mu_{a}I_{a}\left(\frac{N}{K}\right)\\ \frac{dC_{a}}{dt} & =\tau_{j}C_{j}-\theta \mu_{a}C_{a}\left(\frac{N}{K}\right)\\ \frac{dR_{a}}{dt} & =\gamma_{a}I_{a}+\tau_{j}R_{j}-\mu_{a}R_{a}\left(\frac{N}{K}\right). \end{array}$$

The parameters are summarized in the table 1.

We have employed a carrying capacity (a constant K ) approach, which is widely used in ecology [44], to control the unrealistic exponential growth of rodents in each compartment of our model, because of *M. natalenis*’s high intrinsic population growth rate [24,26,27]. Carrying capacity is the maximum population size that an environment can support indefinitely, i.e. the largest number of organisms that can live in a certain place without causing environmental issues such as the exhaustion of available food. The equation


$$N_{t}=\frac{K}{1+e^{a-rt}}$$

represents how a population grows over time. This is known as the integral form. The equation


$$\frac{dN}{dt}=rN\left(1-\frac{N}{K}\right)$$

explains how a population changes over time. This is called the differential form. In these equations, N stands for the number of organisms, r is the rate at which the population grows when there is no competition for resources, t is time and a is a constant of integration defining the position of the curve relative to the origin. In the second equation, the part inside the brackets indicates the potential for growth. When the population is small, this value is close to 1 , leading to rapid growth. As the population approaches the carrying capacity K , this value decreases towards 0 , causing growth to slow down. When the population reaches K , growth stops because the unused growth potential becomes 0 . This means there is feedback in the system and that the population ceases to grow when it reaches its carrying capacity.

In our model, we incorporate the variable K to establish an upper limit on the size of the rodent populations in each compartment. This is achieved by the influence of K on the death rate and the size of each compartment (e.g. S,I,C,R ), which enhances mathematical, as well as the biological and population, stability. It is important to note that we are implementing the carrying capacity effect on the death rate, which differs from the previous differential equation where the expression within the brackets acted on the growth rate. So in our case, the expression within the brackets takes the form $\frac{N}{K}$ , where N represents the total rodent population at time t . Consequently, the death term in our model is expressed as


$$-\mu_{y}X\left(\frac{N}{K}\right)$$

where ${\;}_{y}$ is the age class ${,}_{a}$ or ${,}_{j}$ .

**Figure 2.**
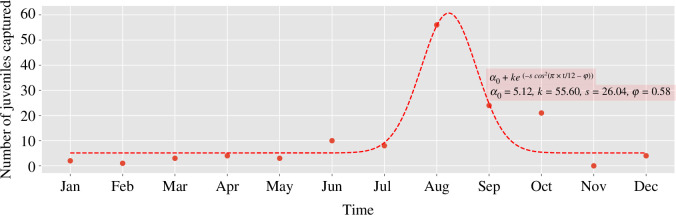
Seasonal breeding pattern of *M. natalensis* overlaid with our fitted birth function curve using data from [26].

The genus *Mastomys* comprises abundant and intensively studied rodents, widespread across sub-Saharan Africa, with *M. natalensis* the best studied [45,46]. However, most data are from regions outside West Africa, which we used to parametrize our model in the absence of the necessary data. Mulungu *et al.* [26] reported that there is a seasonal breeding pattern for *M. natalensis* in Tanzania, East Africa. Although the data from [26] were collected in Tanzania, the multimammate mouse (*M. natalensis*) is a widely distributed species across sub-Saharan Africa, and its reproductive patterns are expected to be influenced by similar environmental cues and seasonal changes. Therefore, while acknowledging potential regional variations, we extrapolated the observed seasonal breeding pattern to West African populations of *M. natalensis*, which serves as the primary reservoir for LASV transmission. This extrapolation is further supported by previous studies (e.g. [4]) that have documented comparable seasonal fluctuations in the reproductive activity of *M. natalensis* in West African regions, lending credence to the broader applicability of the patterns observed by Mulungu *et al.* [26]. In the Tanzanian study, more juveniles were captured in the months of August and September compared with other months, along with a baseline capture number. We extracted data from [26] with the help of the tool WebPlotDigitizer^1^ [47] and fitted the function $\nu (t)=\alpha_{0}+ke^{\left(-s\;cos^{2}(\pi t/12-\varphi )\right)}$ following the approach outlined in [48], except for the baseline birth rate, which is represented by $\alpha_{0}$ , to the data in [26], as shown in figure 2, where $\alpha_{0}$ is the baseline birth rate, k is the birth pulse scaling factor, s is the duration of the birth pulse and φ is the the timing of the peak of the birth pulse.

We need to normalize $\alpha_{0}$ and k to obtain the annual per capita birth rate. Safronetz *et al.* [49] reported the reproductive behaviour of West African *M. natalensis*. In their 12 months breeding program of *M. natalensis*, 90 rodents give birth to 1618 pups. So we can say


(2.2) $$\int_{1}^{12}\left(\alpha_{0}+ke^{\left(-s\;cos^{2}(\pi t/12-\varphi )\right)}\right)dt=\frac{1618}{90/2}$$

assuming an equal number of males and females in the group. Therefore


(2.3) $$\begin{array}{ccc} & 11\alpha_{0}+k\int_{1}^{12}\left(e^{\left(-s\;cos^{2}(\pi t/12-\varphi )\right)}\right)dt=\frac{1618}{45} & \end{array}$$

by substituting the values for s and φ from the fit to the above equation


(2.4) $$k\int_{1}^{12}\left(e^{\left(-31.02\;cos^{2}(\pi t/12-0.59)\right)}\right)dt=\frac{1618}{45}-11\alpha_{0},$$

i.e.


(2.5) $$\begin{array}{ccc} & k\times 1.226=\frac{1618}{45}-11\alpha_{0} & \end{array}$$

or


(2.6) $$k=\frac{\left(\frac{1618}{45}-11\alpha_{0}\right)}{1.226}.$$

This is an equation with two unknowns, so we need to fix one before we can continue. On average, a female *M. natalensis* can give birth to nine pups per litter every 3.25 months [49].

So a female *M. natalensis* can produce an average of $\frac{9}{3.25}=2.775$ offspring per month. Hence $\alpha_{0}$ , the baseline birth rate per capita per month is 2.775 pups.

Therefore


(2.7) $$k=\frac{\left(\frac{1618}{45}-11\frac{9}{3.25}\right)}{1.226}=4.51.$$

Thus, the normalized birth rate is


(2.8) $$\begin{array}{ccc} & \nu (t)=2.77+4.51e^{\left(-31.02\;\cos^{2}(\pi t/12-0.59)\right)} & \end{array}$$

shown in figure 2.

Together, this gives us a parametrized LASV-*M. natalensis* S,I,C,R model with which to perform simulation exercises and test the impact of varying scenarios.

### 2.1. Varying birthing cycles

*Mastomys natalensis* is a widespread species [45], living in a range of environments, including peridomestically [50], and it is a species that responds to environmental factors such as rainfall and associated food availability [5,24,25,28,29,32]. Given this, human interventions such as land use change [30] and climate change impacts on rainfall [31] will impact *M. natalensis*. Climate and land use change can induce either an expansion (widening) or a contraction (narrowing) of the birth pulse (figure 2) in the *M. natalensis* populations as they impact the availability of resources. To simulate or experiment with such changes, we varied the width of the birth pulse by altering the parameter s in the birth pulse function


(2.9) $$\nu (t)=\alpha_{0}+ke^{-s\cos^{2}(\pi t/12-\varphi )}.$$

During this experiment, it is important to ensure that the integral of the birth pulse remains the same as before, which means


(2.10) $$\int_{1}^{365}(\alpha_{0}+ke^{-s\cos^{2}(\pi t/12-\varphi )}),dt=39.214309.$$

This assumption implies that the number of juveniles captured over the year, approximately 39, remains constant even as the capture window changes, with a background birthing rate included so that there are births throughout the year.

Now we can begin experiments, so here we are going to test two different scenarios, which are

**Figure 3.**
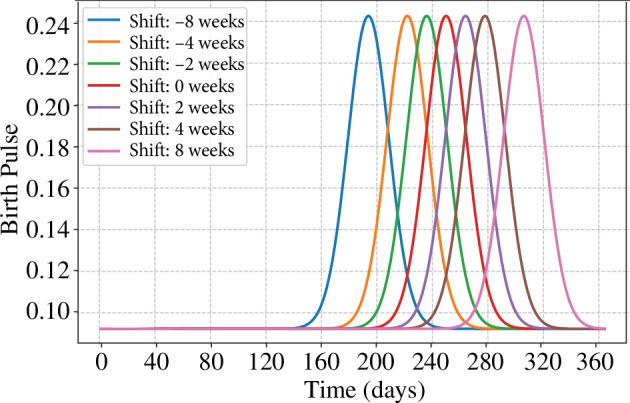
Shifting the seasonal birthing peak.

**Figure 4.**
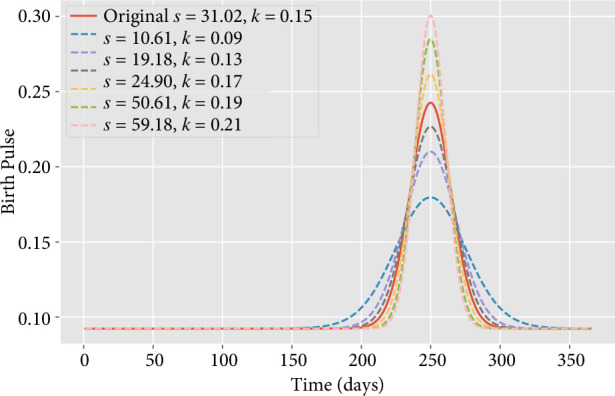
Comparison of the size and synchrony of birth pulses for the original fitted parameters and selected varying parameters with increased or decreased synchrony, but same per capita annual birth rate.

— the impact of shifting the seasonal peak of births by varying φ (figure 3) and

— the impact of widening the birthing season by varying s (figure 4),

which are both potential impacts of varying land use and climate and we can estimate the periodic basic reproductive number ( $R_{p}$ ) and determine the parameters that the model is sensitive to using a sensitivity analysis.

### 2.2. Time-average basic reproduction number

Two primary approaches used for calculating the basic reproductive number in non-autonomous disease transmission periodic systems are the time-average method and the linear operator method [22,51–54]. Mitchell and Kribs [55] conducted a comparison of the two methods, identifying the conditions under which they align. In this work, we use the time-average method to establish the $R_{p}$ of the model (2.1).

When the population is LASV free, we have $I_{j}=C_{j}=R_{j}=I_{a}=C_{a}=R_{a}=0$ , and the model (2.1) has a disease-free equilibrium, denoted $\varepsilon_{0}$ given by


(2.11) $$\begin{array}{ccc} & \varepsilon_{0}=(S_{j}^{*},I_{j}^{*},C_{j}^{*},R_{j}^{*},S_{a}^{*},I_{a}^{*},C_{a}^{*},R_{a}^{*})=(N_{S_{j}},0,0,0,N_{S_{a}},0,0,0), & \end{array}$$

where $N_{S_{j}}$ and $N_{S_{a}}$ are the initial susceptible juveniles and adults.

Hence, linearizing system (2.1) at $\varepsilon_{0}$ , we obtain the following equations:


(2.12) $$\begin{array}{rl} \frac{dI_{j}}{dt} & =(\beta_{ja}I_{a}+\beta_{jj}I_{j}+\beta_{c}(C_{j}+C_{a}))S_{j}^{*}-\tau_{j}I_{j}-\gamma_{j}I_{j}-\theta \mu_{j}I_{j}\left(\frac{N}{K}\right)\\ \frac{dC_{j}}{dt} & =\nu (\rho (I_{a}+C_{a}))-\tau_{j}C_{j}-\theta \mu_{j}C_{j}\left(\frac{N}{K}\right)\\ \frac{dI_{a}}{dt} & =\tau_{j}I_{j}+\left(\beta_{aa}I_{a}+\beta_{ja}I_{j}+\beta_{c}(C_{a}+C_{j})\right)S_{a}^{*}-\gamma_{a}I_{a}-\theta \mu_{a}I_{a}\left(\frac{N}{K}\right)\\ \frac{dC_{a}}{dt} & =\tau_{j}C_{j}-\theta \mu_{a}C_{a}\left(\frac{N}{K}\right). \end{array}$$

Using the notation in [56], the next generation matrix F and V associated with the model (2.1) are given, respectively, by


(2.13) $$\begin{array}{ccc} & \begin{array}{c} F=\left(\begin{array}{cccc} \beta_{jj}S_{j}^{*} & \beta_{c}S_{j}^{*} & \beta_{ja}S_{j}^{*} & \beta_{c}S_{j}^{*}\\ 0 & 0 & 0 & 0\\ \beta_{ja}S_{a}^{*} & \beta_{c}S_{a}^{*} & \beta_{aa}S_{a}^{*} & \beta_{c}S_{a}^{*}\\ 0 & 0 & 0 & 0 \end{array}\right), \end{array} & \end{array}$$

(2.14) $$\begin{array}{ccc} & \begin{array}{c} V=\left(\begin{array}{cccc} \tau_{j}+\gamma_{j}+\theta \mu_{j}(\frac{N}{K}) & 0 & 0 & 0\\ 0 & \tau_{j}+\theta \mu_{j}(\frac{N}{K}) & -\nu (t)\rho & -\nu (t)\rho \\ -\tau_{j} & 0 & \gamma_{a}+\theta \mu_{a}(\frac{N}{K}) & 0\\ 0 & -\tau_{j} & 0 & \theta \mu_{a}(\frac{N}{K}) \end{array}\right). \end{array} & \end{array}$$

$$\begin{array}{rl} R_{p} & =\rho (FV^{-1})\\ & =\frac{KS_{a}^{*}(K^{2}\beta_{aa}\nu (t)\rho \tau_{j}-K^{2}\beta_{c}\nu (t)\rho \tau_{j}-KN\beta_{aa}\mu_{a}\tau_{j}\theta -KN\beta_{c}\mu_{a}\nu (t)\rho \theta -N^{2}\beta_{aa}\mu_{a}\mu_{j}\theta^{2})}{K^{3}\gamma_{a}\nu (t)\rho \tau_{j}-K^{2}N\gamma_{a}\mu_{a}\tau_{j}\theta +K^{2}N\mu_{a}\nu (t)\rho \tau_{j}\theta -KN^{2}\gamma_{a}\mu_{a}\mu_{j}\theta^{2}-KN^{2}\mu_{a}^{2}\tau_{j}\theta^{2}-N^{3}\mu_{a}^{2}\mu_{j}\theta^{3}}, \end{array}$$

where $\rho (FV^{-1})$ is the spectral radius of the next generation matrix $FV^{-1}$ at the disease-free equilibrium $\varepsilon_{0}$ .

Replacing ν(t) by its long-term average [55] we have


$$\begin{array}{c} {}[\nu ]:=\frac{1}{\omega }\int_{0}^{\omega }\nu (t)dt. \end{array}$$

Then, we have the time-average ${\tilde{R}}_{p}$ given by


(2.15) $${\tilde{R}}_{p}=\frac{KS_{a}^{*}(K^{2}\beta_{aa}[\nu ]\rho \tau_{j}-K^{2}\beta_{c}[\nu ]\rho \tau_{j}-KN\beta_{aa}\mu_{a}\tau_{j}\theta -KN\beta_{c}\mu_{a}[\nu ]\rho \theta -N^{2}\beta_{aa}\mu_{a}\mu_{j}\theta^{2})}{K^{3}\gamma_{a}[\nu ]\rho \tau_{j}-K^{2}N\gamma_{a}\mu_{a}\tau_{j}\theta +K^{2}N\mu_{a}[\nu ]\rho \tau_{j}\theta -KN^{2}\gamma_{a}\mu_{a}\mu_{j}\theta^{2}-KN^{2}\mu_{a}^{2}\tau_{j}\theta^{2}-N^{3}\mu_{a}^{2}\mu_{j}\theta^{3}}.$$

### 2.3. Sensitivity analysis

In this section, we explore the significance of model parameters on transmission dynamics of LASV using sensitivity analysis. The sensitivity analysis enables the assessment of proportional change in the basic reproduction number as the model parameter is varied [57]. For the LASV model (2.1), the normalized forward sensitivity indices of the time-average basic reproduction number ${\tilde{R}}_{p}$ with respect to the parameter ζ are calculated using the following:


(2.16) $$\Gamma_{\zeta }^{{\tilde{R}}_{p}}=\frac{\partial {\tilde{R}}_{p}}{\partial \zeta }\times \frac{\zeta }{{\tilde{R}}_{p}},$$

where ζ is any parameter in the expression of ${\tilde{R}}_{p}$ .

We also test the impact of the initial numbers of chronically infected juvenile populations by varying in the initial conditions, shown in the electronic supplementary material, appendix.

## 3. Results

### 3.1. Simulation results

Overall, our model replicates some key features of LASV dynamics in *M. natalensis*. There is a peak of births and viral prevalence in a two- to three-month period, correlating to the months of August and September, with the highest viral prevalence occurring in the juvenile class (i.e. approximately 10% of adults and 56% of juveniles infected). The above observation is consistent with the presence and absence of chronically infected individuals in both adults and juveniles.

**Figure 5.**
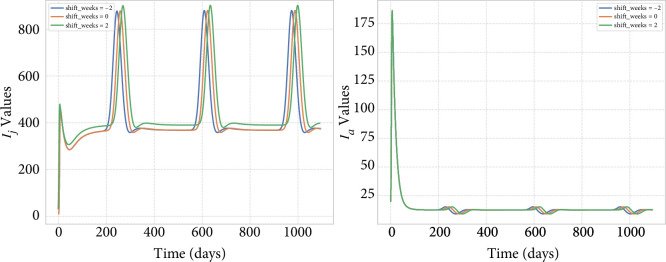
Effect of changing the birth pulse birth timing.

*Viral prevalence and seasonal changes in birthing timing*. Iif changes like climate or land use change move the seasonal peak of birthing (e.g. figure 3), our simulation suggests that this will simply shift the peak of viral prevalence. The results of the simulation are shown in figure 5.

**Figure 6.**
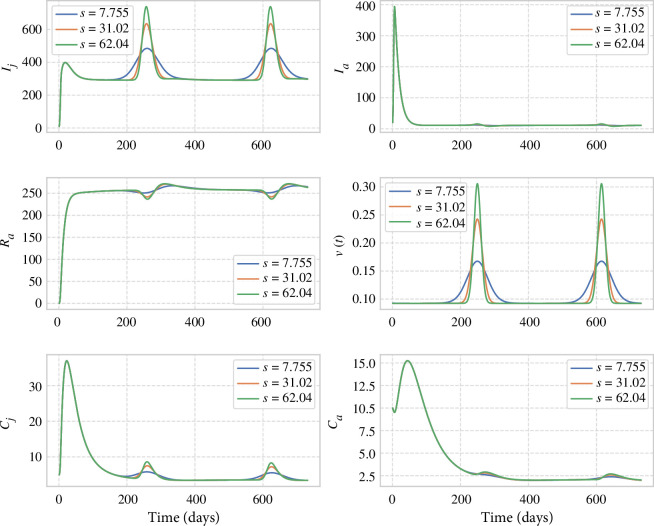
Effect of changing the birth pulse birth width.

*Viral prevalence and seasonal changes in birthing synchrony*. If changes like climate or land use alter the synchrony of the seasonal birth peak (e.g. figure 4), our simulation suggests that this will alter both the timing and height of the peak viral prevalence, primarily in the juvenile population. The impact of changes in birthing patterns is most pronounced in the number of infected juveniles ( $I_{j}$ ), with smaller effects on infected adults ( $I_{a}$ ). Adult chronic infection levels ( $C_{a}$ ) remain relatively stable across different birth synchrony scenarios. The results of the simulation are shown in figure 6.

The impact of the changes in birthing is largely seen in the juvenile, rather than adult classes, with the adult numbers of infected individuals relatively stable (figures 6 and 3).

The impact of varying the initial numbers of chronically infected juvenile populations in the initial conditions is shown in the electronic supplementary material (appendix figures A1 and A2).

### 3.2. Sensitivity analysis

**Figure 7.**
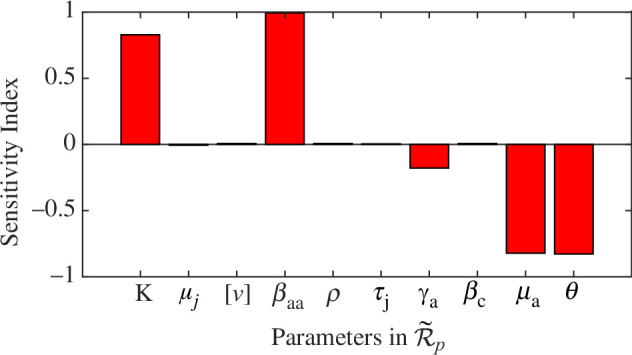
Sensitivity indices of the time-average basic reproduction number ${\tilde{R}}_{p}$ .

The sensitivity indices of ${\tilde{R}}_{p}$ to parameters for the LASV model (2.1), evaluated at the parameter values in table 1, are provided in table 2. The positive sign of the sensitivity index of the time-average basic reproduction number, ${\tilde{R}}_{p}$ , with respect to the model parameters indicates that an increase (or decrease) in the value of each parameter will result in an increase (or decrease) in the basic reproduction number of the disease. It is observed from table 2 that four parameters, K , $\beta_{aa}$ , $\mu_{a}$ and θ , have the greatest impact on ${\tilde{R}}_{p}$ , with a fifth, $\gamma_{a}$ , also contributing. The plot of the sensitivity indices for each parameter in ${\tilde{R}}_{p}$ is shown in figure 7. The positive indices of parameters K and $\beta_{aa}$ show that they have a direct relation with ${\tilde{R}}_{p}$ . For example, an increase or decrease in the value of K by 10% would increase or decrease the value of ${\tilde{R}}_{p}$ by 8.22% . The negative indices of parameters $\mu_{a}$ and $\gamma_{a}$ show that they have an inverse relation with ${\tilde{R}}_{p}$ . Increasing the value of $\mu_{a}$ by 10% would results in 8.22% decrease in the value of ${\tilde{R}}_{p}$ .

**Table 2.** Sensitivity indices of the time-average basic reproduction number with respect to parameters for the LASV model (2.1).

parameter	index	increase or decrease (%)	impact on ${\tilde{R}}_{p}$
K	+0.8282	10	8.22%
$\mu_{j}$	−0.006452	10	0.065%
[ν]	+0.00652	10	0.065%
$\beta_{aa}$	+0.9935	10	9.94%
ρ	+0.00652	10	0.065%
$\tau_{j}$	+0.000415	10	0.0042%
$\gamma_{a}$	−0.1787	10	1.78%
$\beta_{c}$	+0.00651	10	0.065%
$\mu_{a}$	−0.8218	10	8.22%
θ	−0.8282	10	8.28%

**Figure 8.**
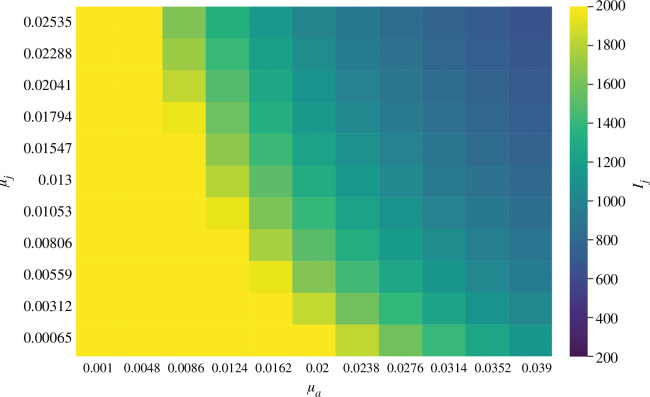
The influence of varying $\mu_{a}$ and $\mu_{j}$ on $I_{j}$ on day 258 of simulations is examined. We have selected a range of 95 variations on either side of the observed values of $\mu_{j}=0.013$ and $\mu_{a}=0.02$ to explore extreme scenarios or situations characterized by high uncertainty surrounding the observed values of $\mu_{a}$ and $\mu_{j}$ .

The results of this sensitivity analysis can be seen in simulations. For example, figure 8 shows the impact of varying $\mu_{a}$ and $\mu_{j}$ on $I_{j}$ .

## 4. Discussion

We developed a SICR model of LASV dynamics in its rodent host, *M. natalensis*. Our simulations suggest that altering rodent dynamics, such as through changing resource availability with climate or land use change, will impact LASV dynamics in two ways. First, by shifting the peak of viral prevalence if seasonality changes, thus impacting the potential risk of LASV in people. Second, by modelling the impacts of shifting synchrony. We note that the infection prevalence is very stable in adults, and the impacts are largely seen among juveniles, potentially helping inform risk mitigation strategies.

Our sensitivity analyses suggest there are four key parameters that impact ${\tilde{R}}_{p}$ : carrying capacity ( K ), adult mortality ( $\mu_{a}$ ), adult-to-adult transmission rate ( $\beta_{aa}$ ) and additional disease-induced mortality ( ω ). Impacting these will probably have the greatest changes on LASV dynamics. A fifth parameter, $\gamma_{a}$ , the infection recovery rate, also has greater impact on ${\tilde{R}}_{p}$ , than the remaining parameters. Notably, carrying capacity and adult mortality are two parameters that can probably be substantially impacted by humans, because, for example, crops might provide resources for *M. natalensis* [25,28] and increase or decrease mortality, along with human activities such as rodent control and killing [17]. Changes in density will also probably impact $\beta_{aa}$ , which itself is dependent on contact rates [58,59]. These responses are probably general too, i.e. in North American deer mouse, *Peromyscus maniculatus*, a reservoir host for numerous zoonotic pathogens common in peridomestic settings, contact rates have been shown to significantly increase in response to feeding [60].

Our findings have similarities and differences to other systems. Our sensitivity analyses are not directly comparable to sensitivity analyses for other recent LASV model with time-dependent parameters that have different model structures and include human transmission [16,22]. However, Ibrahim & Dénes [22] found both reproduction numbers they modelled, a basic $R_{0}$ and time-average basic reproduction number ${\tilde{R}}_{p}$ increase by increasing the transmission rates and rodent birthing rates, the latter differing from our findings. In other systems, sensitivity analyses of filovirus (e.g. Ebola virus, Marburg virus) dynamics and persistence in bat host populations, viruses which also cause zoonotic VHF and disease in people in Africa, suggest that persistence is significantly positively affected by increasing the incubation period, infectious period and overall birth rate, and negatively by increasing synchrony of the birth pulse, rather than carrying capacity or mortality rates [61], suggesting that processes that affect different parameters will probably alter the risk of infection transmission among emerging viruses differently.

Our sensitivity analyses also help us understand which parameters might be important to measure in the field [62]; we used some data from East African *M. natalensis* populations to model LASV, a disease occurring in West Africa, because of a lack of appropriate data. Despite this, our work and that of others suggest in West Africa the *M. natalensis* population dynamic fluctuation depends on seasonally available food, and human activities, habitat or rainfall changes will probably impact these. These studies are important; in Upper Guinea a seven-year rodent control experiment with 10–30 day control periods annually and a single intensive three-month trapping exercise led to a rapid increase in rodents following control efforts with concurrently high LASV infection rates, suggesting density-dependent compensation drove increased viral incidence [17]. Our model supports these field studies, suggesting continuous control to reduce the carrying capacity ( K ) and adult mortality ( $\mu_{a}$ ) might be most successful in reducing ${\tilde{R}}_{p}$ . Figure 8 shows the relative impact of increasing mortality on the infected juvenile classes ( $I_{j}$ ), which have greater prevalence than adults.

We aimed to have a parsimonious, but biologically realistic model that allows us to run the experiments we wanted. We limited our simulations to those models with seasonal forcing, because without seasonal forcing we would expect dampening oscillating dynamics until an endemic state is produced and then risk to humans is owing to seasonally changing contact rates (if they exist) and human behaviours and risk factors, such as immunity. This would be true even in the presence of other dynamic factors, such as maternal antibody, in a deterministic model. We have previously looked at the impact of maternally derived antibodies in other systems, including those with strong seasonality (e.g. [63,64]). In LASV*–M. natalensis* system, it appears that maternally derived antibodies last about 30 days on average. On the whole, including maternally derived antibodies is likely to simply dampen the seasonality due to seasonal forcing due to birthing, thus having an analogous role to decreasing the birthing synchrony which we model.

*Future analyses*. We used a relatively simple model; however, additional details could be added to future models and questions asked. Furthermore, our model is deterministic. LASV is maintained in *M. natalensis* populations, therefore this suffices; however, a stochastic version of this model would help to explore how infection may or may not fade out, with alternative questions, such as below, through stochastic simulations [43,61,65].

There are multiple questions that we did not ask. These include:

— How might an incubation period, or exposed (*E*) class, alter transmission dynamics? There is evidence of a short incubation period for LASV in *M. natalensis*; peak viral RNA was detected in nine tissues (all tested) of experimentally infected mice just days 7 and 14 post-infection [37], so animals are probably infected just days into infection. However, longer incubation periods may alter the dynamics. This may be more important for viral maintenance in the populations in systems with very highly synchronized birthing, as observed in some bat systems [61,64] or, conversely, where there is little seasonality, such in Niamey, Niger, in the Sahel, where *M. natalensis* is mostly associated with people, living mostly indoors [50], because incubation periods may allow a greater probability of viral persistence in a population [61].
— How does vertical transmission of LASV in juvenile rodents impact overall virus prevalence and persistence in the population? The model could examine different rates of (pseudo)vertical transmission, which might facilitate persistence and be important for resurgence of infections following large declines in populations, including following control [17].
— What is the impact of seasonal fluctuations in host population density on LASV transmission dynamics? The model could explore different scenarios with high versus low amplitude population cycles. New datasets for *M. natalensis*, such as a recent 29-year dataset in East Africa, will allow more data-driven analyses to be performed [46] to understand time-varying risk in more detail. Climate change is predicted to impact West Africa in two alternative, but plausible ways, with either decreased rainfall projected in the Gulf of Guinea in spring and the Sahel in summer, or increased summer rainfall over both regions [31]. In years when rainfall was below average and the wet season was short, *M. natalensis* population densities were significantly lower [24]. Moreover, these could also incorporate aspects of control to better understand how different control measures might dampen or even increase the viral incidence in populations, as seen in Guinea, West Africa [17].
— What is the effect of rodent reproductive seasonality and birth pulses on LASV prevalence over time? Related to the three questions above, the model could test shifting or expanding/contracting birth pulse seasons that change through time with varying amplitudes.
— How does acquired immunity following LASV infection in rodents impact transmission dynamics and prevalence? As discussed above, the model could incorporate waning immunity and reinfection of recovered individuals and maternally derived antibodies in the young, as in Mariën *et al*. [23].
— How sensitive are the model predictions to uncertainties in key parameters not included or included in other terms, such as contact rates and transmission probabilities within *β* terms? A sensitivity analysis of a more complex model could identify critical knowledge gaps, along with stochastic models to help understand viral persistence. Common findings across models with different structures strengthen the case for these parameters being important drivers of viral persistence.
— Can the model be expanded to connect LASV dynamics in rodents to human exposure risk? Adding a human component, such as seasonally changing behaviours (and so exposures) could better predict case and outbreak risk [13,21]. Efforts have been made (e.g. [16,22]), but as better data become available, models can help identify key transmission dynamics between and among hosts [9,66].
— How would interventions like rodent control or landscape modification potentially impact LASV transmission in rodent populations and human risk? Adding intervention effects could inform disease control, beyond our findings that carrying capacity (*K*) and adult mortality (*µ_a_*) are key parameters for this system.
— How might new species recently identified as potential hosts impact the viral dynamics and human risk? Recent studies such as [1,3] have identified other rodent species that can be infected with LASV. These are typically even less well studied than *M. natalensis* in West Africa, but future models could include differing host dynamics to better understand if and how these alternative hosts might alter viral persistence and human risk.

Future analyses can use our model to inform field and experimental studies [62], with temporally and spatially varying data particularly useful to further validate the model predictions. Recent analyses with a similar *SEIR*-based model, including the same periodically forced seasonal birthing from [48] and an approximate Bayesian computation scheme to fit the model to the case data from 2018 to 2020 in Nigeria, also showed that the dynamics of the rodent reservoir probably lead to periodic increases in spillover risk [16], but this lacked the rodent data and so further work is needed. Our analyses help determine which parameters of the models outcomes are sensitive to, and so which aspects of the system will lead to changing risk and require better data.

In summary, we developed an SIR -based model of LASV dynamics in its rodent host, *Mastomys natalensis*, with a persistently infected class and seasonal birthing to test how climate and land use changes that impact birthing might alter viral dynamics and, therefore, risk. Our simulations suggest viral dynamics in adults is mainly stable, with greater variation in the young, with likely predictable shifts in rodent birthing timing and synchrony corresponding to viral prevalence shifts. Our ${\tilde{R}}_{p}$ calculation using a time-average method and with a sensitivity analysis show carrying capacity ( K ), the transmission parameter among adults ( $\beta_{aa}$ ) and adult mortality ( $\mu_{a}$ ) are key parameters impacting LASV in ${\tilde{R}}_{p}$ *M. natalensis* most, all of which are impacted by directly and indirectly human activities and interventions.

## Data Availability

All code and data are available at Zenodo [67]. Supplementary material is available online [68].

## Footnotes

^1^
A comprehensive user manual can be accessed through the software’s GitHub page at https://github.com/automeris-io/WebPlotDigitizer
